# Scleral concave pool trabeculectomy combined phacoemulsification in primary open-angle glaucoma with cataract

**DOI:** 10.1186/s12886-020-01500-2

**Published:** 2020-06-09

**Authors:** Xiangxiang Ye, Yongjun Qi, Jianhua Deng, Yang Yang, Ting Mo, Mao Xu, Wanjun Liu

**Affiliations:** Guangdong Hospital of Traditional Chinese Medicine, Zhuhai, China

**Keywords:** Primary open-angle glaucoma, Scleral concave pool trabeculectomy, Cataract, Phacoemulsification, Intraocular pressure

## Abstract

**Background:**

To investigate the safety and efficacy of scleral concave pool trabeculectomy (SCPT) combined phacoemulsification for eyes with coexisting cataract and primary open-angle glaucoma (POAG).

**Methods:**

This was a retrospective, controlled, interventional pilot case series. Thirty patients (30 eyes) were diagnosed with coexisting cataract and POAG between May 2015 and April 2018. Fourteen eyes underwent SCPT combined phacoemulsification were set as the study group, and 16 eyes received conventional phacotrabeculectomy were set as the control group. All patients were followed up for at least 6 months. The preoperative to postoperative changes in IOP, glaucoma medication requirements, BCVA, blebs functions, and adverse events were recorded.

**Results:**

The groups were matched for baseline age, BCVA, IOP and types of IOP-lowering medications (all *P* > 0.05). At 6-month visit, there were no significant difference between control and study group in the improvement of BCVA (0.22 ± 0.24 versus 0.18 ± 0.26, *P* = 0.718), reduction of IOP (− 11.21 ± 8.61 mmHg versus − 9.19 ± 9.18 mmHg, *P* = 0.540) and the number of eyes that needed IOP-lowering medications (2 versus 3, *P* = 0.743). At the last visit, the rate of forming functioning blebs was significantly different between the study and control groups, (92.9% versus 68.7% respectively, *P* = 0.007). In the study group, 5 eyes developed hypotony, and 1 eye showed limited choroidal detachment, whereas in the control group 1 eye developed malignant glaucoma. All adverse events were successfully managed.

**Conclusion:**

The SCPT combined phacoemulsification seems to be a safe and effective alternative to conventional phacotrabeculectomy for patients with POAG and visually significant cataract in the short-term.

## Background

Primary open-angle glaucoma (POAG) is one of the major causes of blindness for elderly people who often suffer from coexisting cataract [1]. In such patients, combined phacoemulsification and trabeculectomy (phacotrabeculectomy) can treat coexisting visually significant cataract and advance glaucoma at the same time [2] which is the preferred surgical management in Chinese elderly patients. Fibroblast proliferation and collagen deposition at filter channel is the most common reason for trabeculectomy failure [3–5]. It is widely accepted that the application of adjunctive antifibrotic agents such as 5-fluorouracil (5-FU) and mitomycin C (MMC) can enhance the success rate of filtration surgery [6, 7], but there was still some surgical fails even with help of adjunctive antifibrotic agents [8]. In addition, during a certain period of time, there was not enough supply of 5-FU and MMC drugs in China because many domestic companies stopped producing them. Therefore, it sparks an emergency requirement for the improvement of trabeculectomy technique.

Improvement of techniques, such as deep sclerectomy (DS) and CO_2_ laser-assisted sclerectomy surgery (CLASS) has reduced the complication rates in treating open angle glaucoma [9–11]. Contrary to trabeculectomy, DS, which does not require penetrating the anterior chamber, has been reported to improve the safety of glaucoma surgery with high success rate, but it requires considerable technical skills to preserve the integrity of the trabeculo-Descemet’s membrane (TDM) [9, 12–14]. Recently, a newly developed technique, CLASS, which performs deep sclerectomy down to the TDM by CO_2_ laser application on the scleral bed after a half thickness scleral flap is created, has been reported as a feasible and apparently safe procedure [11, 15]. However, the IOPtiMate system used in the CLASS procedure is too expensive for its wide spread application in developing countries such as China. Furthermore, CLASS technique is a non-penetrating technique which requires a long learning curve.

To our knowledge, a reservoir in the CLASS procedure allowing the percolated aqueous accumulation may help strike a balance between the production and outflow of aqueous humor, which has the advantages of keeping the filtering path unobstructed and reduces the postoperative complication of filtering surgery. Therefore, we developed a penetrating surgery technique to add a scleral concave pool manually in the trabeculectomy procedure which needs shorter learning curve than non-penetrating technique. In our retrospective study, we aimed to investigate the safety and efficacy of scleral concave pool trabeculectomy surgery (SCPT), performed in conjunction with phacoemulsification, for treating eyes with coexisting cataract and POAG.

## Methods

In this retrospective study, we reviewed the operations performed by one surgeon (Yongjun Qi) at the Guangdong Hospital of Traditional Chinese Medicine, Zhuhai, China between May 2015 and April 2018. All patients considered in this study had coexisting cataract and primary open angle glaucoma. Fourteen eyes (14 patients) underwent SCPT combined phacoemulsification were set as the study group, and 16 eyes (16 patients) received conventional phacotrabeculectomy were set as the control group. Patients were excluded from this study if any of the following conditions exists: gonioscopically narrow or closed angle, history of any vitreo-retinal surgery, fundus diseases (such as age-related macular degeneration, diabetic retinopathy, and retinal detachment), inflammatory eye disorders, or any other potential cause of vision loss. All patients received regular follow-up examinations for at least 6 months.

### Intervention

All procedures followed the tenets of the Declaration of Helsinki. This study obtained approval from the ethical committee of Guangdong Hospital of Traditional Chinese Medicine, Zhuhai, China. No informed consent was obtained due to the retrospective nature of this study.

**Fig. 1:**
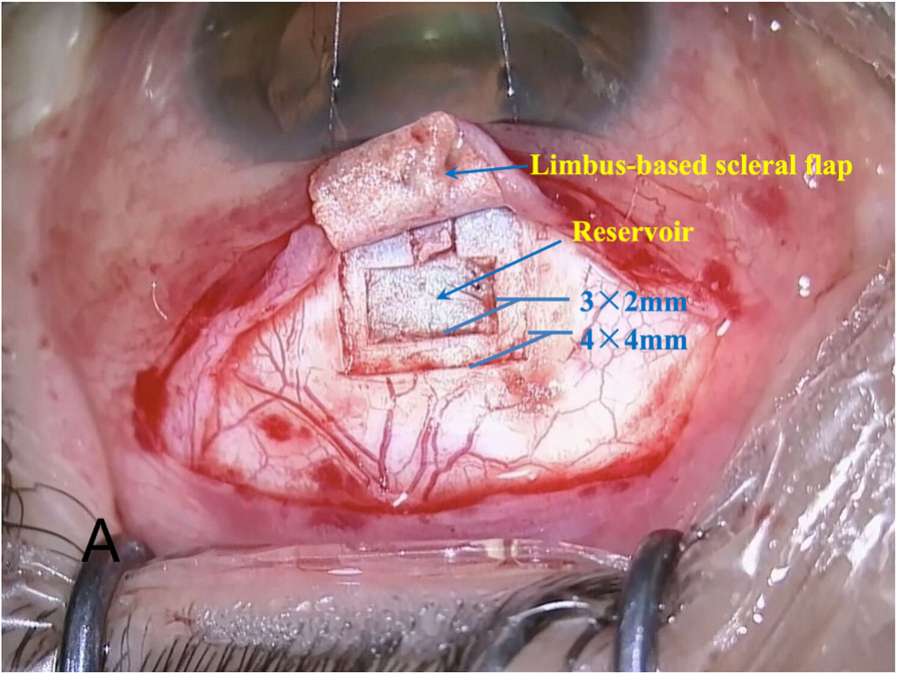
Sketch map of scleral concave pool trabeculectomy surgery design

In both groups, all surgery procedures were performed under peribulbar anesthesia. Before filtration surgeries, phacoemulsification and intraocular lens implantation were performed. A fornix-based conjunctival flap was dissected in the superior quadrant, and the sclera was exposed. A 4 × 4 mm limbus-based scleral flap was then created. For the SCPT procedure, a reservoir measuring about 3 × 2 mm was created over the posterior scleral bed by a surgical blade (Fig. 1). The deeper sclera and the root of Schlemm canal were excised through a convex groove. The superficial scleral flap was closed with 10–0 nylon sutures, and the conjunctival wound was sutured with 10–0 nylon sutures. For the conventional phacotrabeculectomy, no reservoir was further performed. The superficial scleral flap and the conjunctival wound were sutured straightly through the same way with 10–0 nylon sutures. Postoperative management in both groups included overnight patching, topical levofloxacin 5 mg/ml and dexamethasone 1 mg/ml for 4 weeks. Glaucoma medications were administered if necessary.

Postoperatively, all eyes received topical tobramycin and dexamethasone eye drops every 4 h for 1 week and tobramycin and dexamethasone eye ointment every night for 1 week, with gradual tapering over the next 4 weeks. Glaucoma medications were administered if IOP > 21 mmHg. An additional trabeculectomy would be required, if the IOP remained uncontrolled even after maximally-tolerated glaucoma drugs, systemic anti-inflammatory or bleb manipulation were offered.

### Outcome measures

Demographics, systemic diseases, ophthalmic history, topical and systemic medications before the operation were collected. Ocular examinations conducted at baseline and at each follow-up visit (1 day, 1 week, 1 month and 6 months) including best corrected visual acuity (BCVA) with Snellen chart, Goldmann applanation tonometry, slit-lamp examination, gonioscopy, and fundus examination were reviewed. Filtering blebs were categorized as 4 types according to the Kronfeld’s classification [16]. In brief, thin and polycystic blebs with transconjunctival flow of fluid were classified as Type I bleb, other flatter, thicker, and more diffuse blebs with a relatively vascular appearance as Type II bleb, failed bleb in which conjunctiva is scarred to underlying episclera as Type III bleb, and encapsulated bleb with characteristic vascular, dome-shaped, cyst-like appearance as Type IV bleb. Procedure related complications and number of IOP lowering medications administrated postoperatively were recorded. In the current study, completed success meant postoperative IOP remained stable and was less than 21 mmHg without glaucoma medication, while qualified success meant postoperative IOP remained stable and was less than 21 mmHg with glaucoma medication.

### Data analysis

Data were first tested for normality using Sample Kolmogorov-Smirnov test. Variable confirming to normal distribution were summarized as means ± standard deviation (SD) except when stated otherwise. Comparisons of normally distributed variables between the groups were conducted using independent samples t test, or with nonparametric test if variables are not normally distributed. Changes in BCVA and IOP from baseline to four follow-up visits between the 2 groups were examined using a repeated measure ANOVA. The rate of eyes did not require any IOP-lowering medication and postoperative bleb manipulation was plotted using the Kaplan-Meier method and analyzed using the log-rank test. The number of functioning blebs between the two groups at each visit was compared using Mann-Whitney U test. Statistical analysis was performed using SPSS Version 16.0 (SPSS 16.0, Inc., Chicago, IL). Significance was determined as *P* < 0.05 at two tails.

## Results

**Fig. 2:**
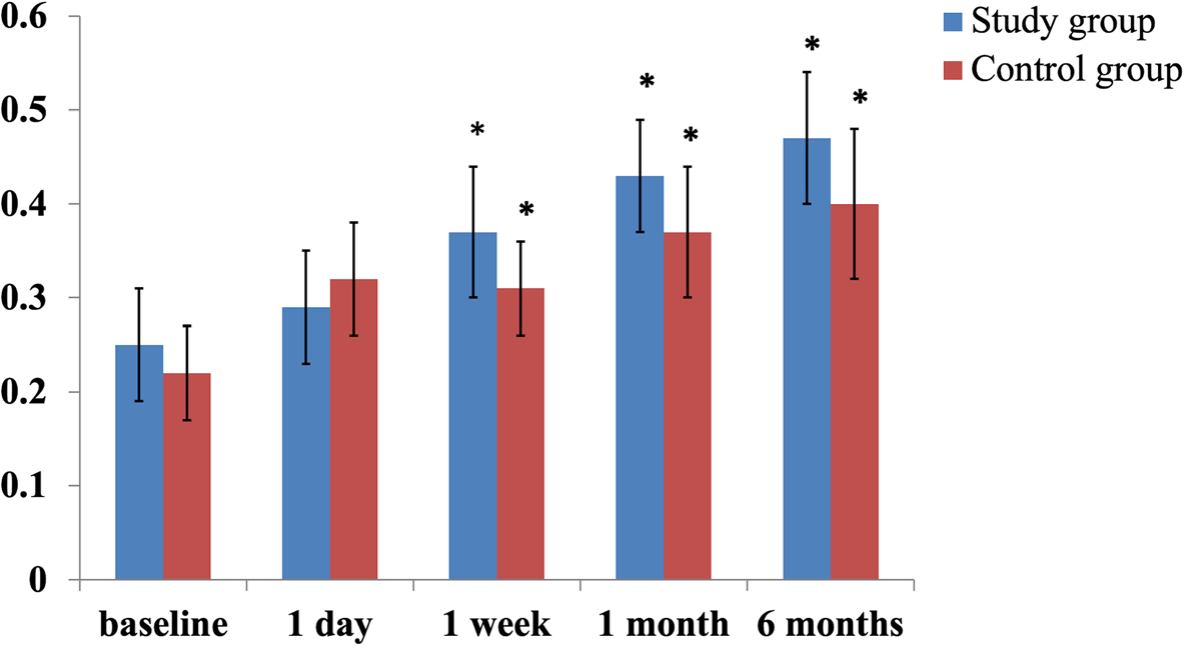
Change in best corrected visual acuity after surgery. *: Significantly different from baseline (*P* < 0.05). Nevertheless, the magnitude of visual improvement was comparable between both groups at each visit (all *P* > 0.05)

As shown in Table 1, there was no significant difference in the mean baseline age, gender, BCVA, IOP and number of medications used between the two groups (all *P* > 0.05). Compared to baseline, postoperative BCVA significantly improved at 1-week, 1-month and 6-month visit in both groups (all *P* < 0.05), but the magnitude of visual improvement was comparable between both groups at each visit (all *P* > 0.05) (Fig. 2).

**Table 1:** Baseline variables characteristics of between the groups

Variables	Study group (*N* = 14)	Control group (*N* = 16)	*P*-values
Age (years)	68.50 ± 7.53	70.81 ± 6.55	0.376
Gender (female/male)	6/8	8/8	0.696
BCVA	0.25 ± 0.22	0.22 ± 0.19	0.671
IOP	24.64 ± 8.56	23.19 ± 8.45	0.644
Medications	3	3	0.719

*N* Number of eyes; *BCVA* Best corrected visual acuity; *IOP* Intraocular pressure; *P* < 0.05 at two tails was considered to be statistically significant.

### Intraocular pressure and number of medications

**Fig. 3:**
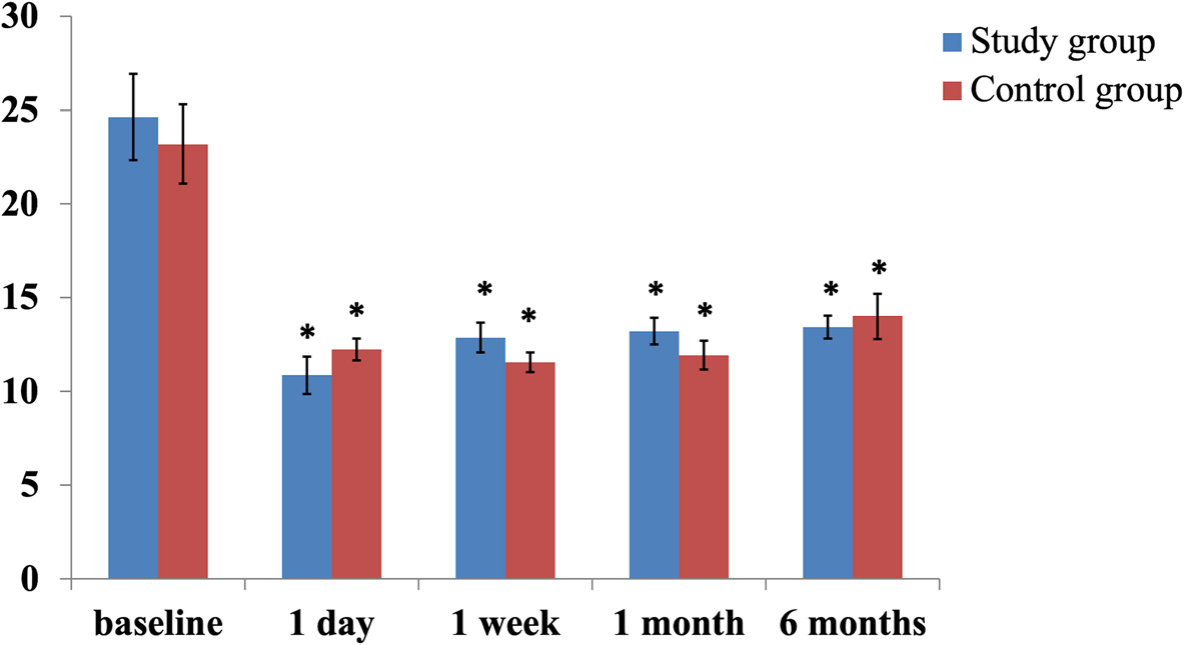
Change in intraocular pressure after surgery. *: Significantly different from baseline (*P* < 0.05). Nevertheless, there was no significant difference in the magnitude of IOP reduction between the 2 groups at the each visit (all *P* > 0.05)

**Fig. 4:**
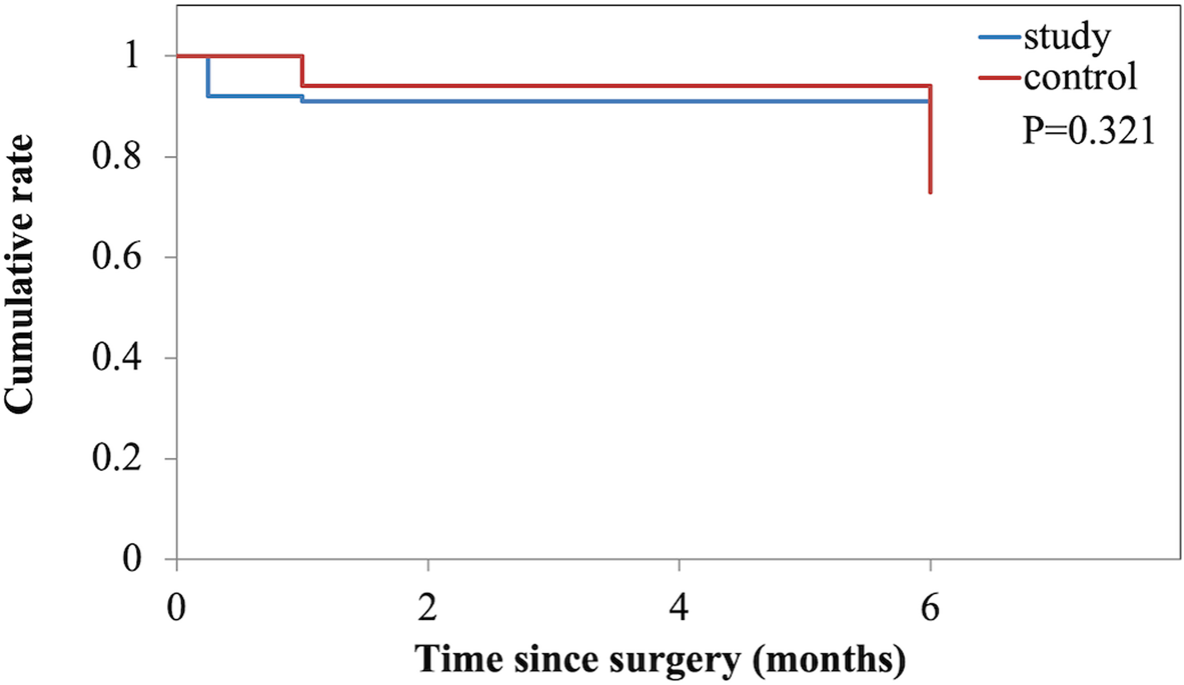
Kaplan-Meyer survival curve for the rate of eyes did not require any IOP-lowering management

Figure 3 shows the IOP changes in the two groups during the follow-up. In both groups, the IOP significantly decreased from baseline at each visit. Mean reduction of IOP was − 11.21 ± 8.61 mmHg in the study group and − 9.19 ± 9.18 mmHg in the control group at the last visit. There was no significant difference in the magnitude of IOP reduction between the 2 groups at the each visit (all *P* > 0.05). During the follow-up, the number of eyes did not require any IOP-lowering medication was 12 (85.70%) and 11 (68.75%) in the study and control groups, respectively. The number of eyes received ophthalmic solution to lower the IOP was 2 (14.30%) and 3 (18.75%) in the study and control groups, respectively. In addition, no eye needs postoperative bleb manipulation in the study group, whereas 2 eyes required postoperative bleb manipulation in the control group. Figure 4 show Kaplan-Meier curve for the rate of eyes did not require any IOP-lowering medication and postoperative bleb manipulation.

### Blebs

At the 1-month visit, all eyes showed functioning blebs in both groups. At the last visit, 13 eyes had functioning blebs, and only 1 eye had a Type III bleb in the study group, whereas 5 eyes had failed blebs (3 Type III and 2 Type IV) in the control groups. At the last visit, rate of forming functioning blebs was 92.9 and 68.7% in the study and control groups respectively, with a significant inter-group difference (*P* = 0.007).

### Complications

As for adverse events related to the procedure and their management, in the study group, 4 eyes that developed hypotony with iris adhesion at 3 days post operation were successfully managed with mydriasis of atropine and tensioning the transconjunctival scleral flap sutures. One eye that showed hypotony and limited choroidal detachment required systemic anti-inflammatory treatment, cycloplegia by atropine 1% eye drops and tensioning the transconjunctival scleral flap sutures. In the control group, 1 eye that developed malignant glaucoma at 2 weeks post operation was successfully managed with Nd:YAG laser, cycloplegia by atropine 1% eye drops, 250 ml of mannitol 20% intravenous infusion, and systemic anti-inflammatory treatment.

### Success rate

At the end of the 6-month period, SCPT combined phacoemulsification had a higher qualified successful rate than that of the control group (100.00% vs. 87.5%, *P* < 0.01). Completed success rate was significantly higher in the study group after 6-month intervention (study: 85.70%, control: 68.75%, *P* = 0.027), with more patients underwent SCPT combined phacoemulsification meeting the complete success criteria. Postoperative IOP was still over 21 mmHg in 2 eyes of the control groups (24 mmHg and 25 mmHg, respectively) even after 3 kinds of glaucoma drugs and bleb manipulation were provided.

## Discussion

In the current study, SCPT combined phacoemulsification was comparable to conventional phacotrabeculectomy in terms of lowering IOP and the number of medications over the first 6-month period but has higher rate of forming functional blebs. Patient undergoing SCPT combined phacoemulsification may be easier to develop hypotony in the early stage, but it could recover after some appropriate management.

Lowering of IOP remains the main therapeutic strategy in the treatment of glaucoma, and trabeculectomy is the most widely applied filtration surgical procedure for achieving a target IOP, with up to 90% of long term success to maintain vision-related quality of life [17–20]. The main purpose of filtration surgery is to lower the IOP by creating a path for more efficient drainage of the aqueous humor from the anterior chamber to the subconjunctival space. Therefore, maintaining the filtering path unobstructed is of critical importance. However, trabeculectomies are often complicated with wound healing, postoperative fibrosis, and production of inflammatory mediators which cause bleb failure and subsequent closure of the filtering route [5, 21]. 5-min exposures of Mitomycin C during the procedure was insufficient in preventing the closure of filtering route and the failures of long term control of IOP due to the subconjunctival and scleral fibroblast proliferation [22].

In the current study, both procedures had similar effects on reducing IOP and the number of medications used over the 6-month follow-up period, but both the complete (85.70% vs. 68.75%, *P* = 0.027) and qualified success rate (100.00% vs. 87.50%, *P* < 0.01) were significantly higher in the study group compared to the control group. In terms of blebs, the rate of forming functioning blebs in the study group was also significantly higher than that in the control group (92.9% vs. 68.7%, *P* = 0.007). All these results may suggest a better efficacy for the long term reduction in IOP during the procedure of SCPT combined phacoemulsification compared to conventional phacotrabeculectomy, but further work is needed to confirm the increased efficacy by reviewing long term follow up.

SCPT, as a conventional filtering surgery, have some unavoidable early complications such as postoperative anterior chamber inflammation [23], bleb leaks [24], intraocular hypotony [25], and choroidal effusion etc. [26] all may threaten the visual acuity (VA). In the current study, both surgery procedures had similar effects on the improvement of postoperative VA, even though the postoperative complications were different between them. At 2 weeks after conventional phacotrabeculectomy, 1 eye developed malignant glaucoma, which was successfully managed with some appropriate treatment. In the SCPT combined phacoemulsification group, 5 eyes developed hypotony, but all of them were successfully managed by appropriate intervention. Overall, this modification of conventional filtering surgery seemed to be as safe as the conventional phacotrabeculectomy at least over the early 6-month period, but its long-term safety still needs investigation by further studies.

The CLASS procedure, which showed a similar qualified success rate and reduction in medications compared to conventional trabeculectomy, may be an alternative to trabeculectomy when considering the postoperative complications [11, 15, 27, 28]. During the CLASS procedure, a reservoir is formed by using the CO_2_ laser to ablate the scleral tissue [11]. A reservoir allowing the percolated aqueous accumulation may have the advantage of keeping the filtering path unobstructed by the following pathways: 1) Filling the bleb with aqueous humor all the time which then help maintain the tension of bleb; 2) Help regulate the flow of liquids so that when the flow speed is low in the drainage, the accumulated aqueous humor in the reservoir can offer a replenishment of liquid; 3) Keeping the path filled with liquid flow can help wash away inflammatory factors. The CO2 laser, which can be largely absorbed by water, has little direct effect on the angle tissue when the aqueous starts to percolate [11, 15], However, the IOPtiMate system used in the CLASS procedure is too expensive for its wide spread application in developing countries such as China. Thus, by adopting the advantage of the CLASS procedure, we decided to manually create the reservoir by surgical blade during the filtering surgery. This procedure needs considerable technical skills, but all the surgery procedures were conducted by one experienced surgeon which could help reduce bias of the result. According to previous studies, CLASS, as a nonpenetrating filtration surgery, may be an alternative to trabeculectomy, especially at the earlier glaucoma stage, considering its more attractive complications profile. However, CLASS is less effective than trabeculectomy and cannot be applied to many conditions such as the primary narrow and occludable angles or secondary glaucoma with the presence of peripheral anterior synechia [11, 15]. However, according to the result of this study, by adopting the advantages of CLASS to penetration filtration surgery, SCPT combined phacoemulsification has comparable or even superior efficiency in reducing the IOP than conventional phacotrabeculectomy.

There were some limitations in this study. Firstly, the sample size was relative small, but it may be unsuited to conduct this procedure for too many patients at this stage since it is a new, untested technique. Secondly, the follow-up time was relative short, but its short-term efficacy and safety which was the main purpose of this study could provide some positive implication for its long-term investigation. Thirdly, the procedure of SCPT may require considerable technical skills to form a reservoir by surgical blade, but it could be conquered by more practice. Finally, this is a pilot study with retrospective design and the absence of randomization.

## Conclusions

In conclusion, by applying the advantage of CLASS to conventional trabeculectomy, SCPT combined phacoemulsification showed a similar efficacy in lowering IOP compared to phacotrabeculectomy, but it increased the success rate of forming functional blebs which provides a better implication for the long-term reduction in IOP. Eyes in the early post-operative period may have a tendency to develop hypotony, but it can be successfully managed or theoretically recover after a longer follow-up time. Due to the recentness of the procedure’s invention, the indication of SCPT combined phacoemulsification and the longevity of post-operative IOP outcome remains unknown. Therefore, the revision of our new trabeculectomy technique is still necessary. In addition, we suggest that further randomized prospective studies should compare this new surgery procedure to modern filtration surgery in many conditions, including primary open angle glaucoma, primary angle closure glaucoma or secondary glaucoma etc., with lager sample size and longer follow-up time to monitor its safety, long-term efficacy and indications.

## Data Availability

The datasets generated during and/or analyzed during the current study are available in the repository of Baidu cloud disk, [PERSISTENT WEB LINK TO DATASETS: https://pan.baidu.com/s/1bQBIFqfXHwShgXg7EkYOvg. Fetch Code: hp95].

## Abbreviations

SCPT: Scleral concave pool trabeculectomy
POAG: Primary open angle glaucoma
5-FU: 5-Fluorouracil
MMC: Mitomycin C
DS: Deep sclerectomy
CLASS: CO_2_ laser-assisted sclerectomy surgery
TDM: Trabeculo-Descemet’s membrane
BCVA: Best corrected visual acuity
IOP: Intraocular pressure
